# The important role of NLRP6 inflammasome in *Pasteurella multocida* infection

**DOI:** 10.1186/s13567-022-01095-0

**Published:** 2022-10-12

**Authors:** Xingping Wu, Zheng Zeng, Hongliang Tian, Lianci Peng, Dongyi Xu, Yu Wang, Chao Ye, Yuanyi Peng, Rendong Fang

**Affiliations:** 1 Joint International Research Laboratory of Animal Health and Animal Food Safety, College of Veterinary Medicine, Southwest University, Chongqing, 400715 China; 2 Chongqing Centers for Disease Control and Prevention, Chongqing, 401120 China; 3 Immunology Research Center, Medical Research Institute, Southwest University, Chongqing, 402460 China; 4 Chongqing Key Laboratory of Herbivore Science, Chongqing, 400715 China

**Keywords:** *Pasteurella multocida*, NLRP6 inflammasome, IL-1β, NLRP3

## Abstract

*Pasteurella multocida* (*P. multocida*) can cause severe respiratory disease in cattle, resulting in high mortality and morbidity. Inflammasomes are multiprotein complexes in the cytoplasm that recognize pathogens and play an important role in the host defense against microbial infection. In this study, the mechanism of *P. multocida*-induced NLRP6 inflammasome activation was investigated in vitro and in vivo. Firstly, *P. multocida* induced severe inflammation with a large number of inflammatory cells infiltrating the lungs of WT and *Nlrp6*^−/−^ mice. *Nlrp6*^*−/−*^ mice were more susceptible to *P. multocida* infection and they had more bacterial burden in the lungs. Then, the recruitment of macrophages and neutrophils in the lungs was investigated and the results show that the number of immune cells was significantly decreased in *Nlrp6*^*−/−*^ mice. Subsequently, NLRP6 was shown to regulate *P. multocida*-induced inflammatory cytokine secretion including IL-1β and IL-6 both in vivo and in vitro while TNF-α secretion was not altered. Moreover, NLRP6 was found to mediate caspase-1 activation and ASC oligomerization, resulting in IL-1β secretion. Furthermore, NLRP6 inflammasome mediated the gene expression of chemokines including CXCL1, CXCL2 and CXCR2 which drive the activation of NLRP3 inflammasomes. Finally, NLRP3 protein expression was detected to be abrogated in *P. multocida*-infected *Nlrp6*^*−/−*^ macrophages, indicating the synergic effect of NLRP6 and NLRP3. Our study demonstrates that NLRP6 inflammasome plays an important role in the host against *P. multocida* infection and contributes to the development of immune therapeutics against *P. multocida*.

**Supplementary Information:**

The online version contains supplementary material available at 10.1186/s13567-022-01095-0.

## Introduction

*Pasteurella multocida* (*P. multocida*) is gram-negative bacteria, causing many economically important diseases in a wide range of domestic and wild animals with high mortality and morbidity, such as hemorrhagic septicemia, atrophic rhinitis, and fowl cholera [1]. So far, it has been identified that *P. multocida* has five serotypes including serotype A, B, D, E and F, of which serotype A of *P. multocida* is considered as one of the most common pathogens to cause pneumonia and bovine respiratory disease complex [2]. Furthermore, several virulence factors of *P. multocida* such as the capsule, lipopolysaccharides, and the neuraminidase (sialidase), have been identified to contribute to the pathogenesis of the respiratory disease [3]. Despite the importance of *P. multocida* pathogenesis, *P. multocida* invasion and interactions with the host are still limited and poorly understood.

In order to respond to microbial infection, the host innate immune system is activated by the interactions of immune cells and pathogens. The initiation of the innate immune response depends on the activation of pattern recognition receptors (PRR) including Toll-like receptors (TLR) and Nod-like receptors (NLR) that sense pathogen-associated molecular patterns (PAMP) and damage-associated molecular patterns (DAMP) [4]. Inflammasomes are members of the NLR family and play an important role in the innate immunity against microbial infections. So far, a number of protein receptors have been confirmed to assemble inflammasomes, such as the nucleotide-binding oligomerization domain (NOD), leucine-rich repeat sequence (LRR) protein (NLR) family members NLRP1, NLRP3, NLRP6, NLRP7, NLRC4, and the proteins absent in melanoma 2 (AIM2) [5].

The role of NLRP3 inflammasome in response to microbial infection has been extensively studied. NLRP6 inflammasome is a relatively new member of the NLR family and the detailed role of NLRP6 in response to different microbial infections is yet to be established. Similarly to the activation of NLRP3, PAMP or DAMP-induced activation of NLRP6 recruits caspase-1 or caspase-11 via the adaptor apoptosis-associated speck-like protein (ASC), leading to inflammatory response with the secretion of IL-1β and IL-18 [6, 7]. The role of NLRP6 in the host defense acts differently under different microbial infections or disease conditions. For example, NLRP6 plays an important role in protecting the host against bacterial or viral infections in the intestine where it is highly expressed. However, *Nlrp6*^*−/−*^ mice had higher survival rates and lower bacterial burden in the lungs against *Staphylococcus aureus* and *Streptococcus pneumoniae* through reduced recruitment of macrophages and neutrophils, thereby decreasing clearance of bacteria, indicating that NLRP6 serves as a negative regulator in pulmonary infections [8, 9]. *P. multocida* as Gram-negative bacteria also cause severe pulmonary infection. Transcriptomic analysis on *P. multocida-*infected murine lungs shows multiple genes were involved in *P. multocida-*induced immune response [10]. In addition, our previous study has shown that *P. multocida* could be recognized by NLRP3 and induced IL-1β secretion. Nevertheless, whether NLRP6 is involved in the regulation of *P. multocida*-induced immune response and the host defense role of NLRP6 in response to *P. multocida* is still unknown.

In this study, we investigated the mechanism of *P. multocida-*induced NLRP6 activation in primary mouse peritoneal macrophages and the role of NLRP6 against *P. multocida* in vivo. The results show that NLRP6 regulates recruitment of neutrophils and macrophages in vivo and the generation of NET as well as the secretion of IL-1β during *P. multocida* infection. Furthermore, *P. multocida* induces the formation of ASC speck which combines with NLRP6 protein to completely activate NLRP6 inflammasome. In addition, NLRP6 also regulates the expression of chemokines CXCL1, CXCL2, CXCR2 and NLRP3 in *P. multocida*-infected macrophages, which in turn modulates the activation of NLRP3 inflammasome. Our study provides useful information about *P. multocida*-induced innate immune response, contributing to the development of vaccines against *P. multocida* infection in the future.

## Materials and methods

### Mice

C57BL/6 (WT) mice were purchased from the Chongqing Academy of Chinese Material Medical (Chongqing, China). *Nlrp6*^*−/−*^ mice were kindly provided by Feng Shao from the National Institute of Biological Sciences (Beijing, China). All gene knockout mice were on the C57BL/6 background and were maintained under specific pathogen-free (SPF) conditions. This study was approved by the Institutional Animal Care and Use Committee of Southwest University (IACUC-2019-0508-02).

### Bacterial strains and growth conditions

The highly virulent bovine *P. multocida* capsular type A isolate PmCQ2 (GenBank accession number: LIUN00000000) was isolated from the lungs of calves with pneumonia in Chongqing, China [11] and stored at −80 °C. Firstly, the bacteria were incubated on Martin’s agar plates at 37 °C for 18–24 h. Then, the single colony was incubated in 5 mL RPMI 1640 medium (Gibco, USA) supplemented with 10% FCS (Gibco, USA) at 37 °C for 12 h. Bacterial concentration was determined by colony counting, and the bacteria was diluted with cell culture medium at the indicated concentration for experimental use.

### Preparation of mouse primary peritoneal macrophages and neutrophils

Mice were injected intraperitoneally (i.p.) with 4% thioglycolate broth (Eiken, Japan). After 4 h and 3 days, neutrophils and peritoneal exudate cells (PEC) were collected, respectively. The mice were anesthetized with ether and then 5 mL of RPMI 1640 medium was sucked into the abdominal cavity. After shaking from side to side, the medium containing cells was slowly sucked out. The PEC and neutrophils were suspended in RPMI 1640 medium containing 10% FCS. The cells were counted with a hemocytometer and seeded into each well at 2 × 10^5^ cells/well for 48-well plates or 1.0 × 10^6^ cells/well for 12-well plates. These cells were maintained at a humidified 37 °C incubator with 5% CO_2_. After 2 h incubation, the nonadherent cells were removed and the adherent cells were used for assays described below. More than 95% of the adherent PEC were F4/80-stained positive cells as determined by flow cytometry, and more than 90% of the nonadherent neutrophils were Ly-6G/Ly-6G (Gr-1)-stained positive cells as determined.

### *P. multocida* infection in macrophages and neutrophils

Macrophages and neutrophils were seeded into each well at 2 × 10^5^ cells/well for 48-well plates or 1.0 × 10^6^ cells/well for 12-well plates. Then, Macrophages were infected with *P. multocida* at a multiplicity of infection (MOI) of 1 for 9 h, and subsequently 100 µg/mL ciprofloxacin (Solarbio, Chongqing, China) were added for another 15 h. Neutrophils were infected with *P. multocida* (MOI = 1) for 3 h. After infection, the supernatants and cell lysates were collected for the assays described below.

### *P. multocida* infection in vivo

WT and *Nlrp6*^*−/−*^ mice were anesthetized by intraperitoneal (i.p.) injection with 33.5 mg/kg sodium 1.5% pentobarbital (MREDA, Beijing, China) for anesthesia, and then intranasally infected with 1000 CFU. After 48 h infection, bronchoalveolar lavage fluid (BALF) and peripheral serum were collected to measure cytokine expression. After 3 h, 6 h, 9 h, 24 h, the total RNA of lung tissues from infected and uninfected mice were also extracted to determine chemokine expression. After 48 h, all the mice were euthanized by intraperitoneal (i.p.) injection with 1.5 mL/kg sodium 5% pentobarbital and then lung tissues from infected WT and *Nlrp6*^*−/−*^ mice were collected and homogenized with PBS. Then, the total number of bacteria in the lungs were counted by colony counting. Since the infection time did not exceed 48 h and the bacterial concentration used in this experiment was only 1000 CFU, the possibility of long-term pain in the experimental mice was low; these animals were used for experimental dissection before irreversible and fatal symptoms appeared.

### Hematoxylin and eosin staining sections (H&E stain)

WT and *Nlrp6*^*−/−*^ mice were anesthetized and infected with *P. multocida* for 48 h. After infection, mice were euthanized and dissected to remove lung tissue. Lung tissues were fixed in 10% formaldehyde followed by dehydration and then enclosed in paraffin. The tissues were sectioned and stained with hematoxylin and eosin(H&E) prior to microscopic examination.

### Enzyme-linked immunosorbent assay (ELISA)

Samples were collected and not diluted. Then, cytokines were determined by ELISA according to the manufacturer’s instructions. Mouse ELISA kits used in this study contained TNF-α (254,425–004), IL-6 (279,559–005), IL-1β (267,273–010), and were purchased from Thermo Fisher Scientific (CA, USA). The standard curve range of IL-6 ELISA kit is 4–500 pg/mL and the standard curve range of TNF-α and IL-1β ELISA kit is 8–100 pg/mL.

### ASC oligomerization

Macrophages were cultured on 12-well plate sand infected with *P. multocida* as described above. After incubation, the cells were washed three times with cold PBS and the cells were lysed with cold PBS containing 0.5% Triton X-100. Cell lysates were centrifuged at 13 000 rpm for 15 min at 4 ℃ to obtain the cell pellets. The pellets were washed twice with cold PBS and suspended in 200 μL PBS. The resuspended pellets were cross-linked with 2 mM fresh disuccinimidyl suberate (DSS) at 37 ℃ for 30 min and then the pellets were centrifuged at 13 000 rpm for 15 min at 4 ℃. Finally, the cross-linked pellets were dissolved in 30 μL 1 × SDS-PAGE sample loading buffer and samples were boiled for 5 min before the western blot analysis.

### Western blot analysis

Cells were cultured in a 12-well plate and infected with *P. multocida* as described above. After incubation, the supernatants of PEC and neutrophils were collected and cells were lysed with radio‐immunoprecipitation assay (RIPA) buffer (Beyotime, Beijing, China). These samples were concentrated with 20% (w/v) trichloroacetic acid (TCA) and the concentration was determined using a BCA protein detection kit (Beyotime). The supernatants and cell lysates were separated by a 10–15% SDS-PAGE gel and subsequently transferred to polyvinylidene difluoride (PVDF) membranes. The membranes were immunoblotted with indicated antibodies (Abs) containing mouse anti-mouse caspase1-p20 Ab (AG20B-0042) (AdipoGen, San Diego, USA), goat anti-mouse IL-1β Ab (AF-401-NA) (Bioss, Beijing, China), rabbit anti-mouse gasdermin D (GSDMD) Ab (ab209845) (Abcam, Cambridge, UK), rabbit anti-mouse ASC Ab (67,824) (Cell signaling technology, Danvers, MA, USA), mouse anti-rabbit GAPDH Ab (AG019-1) (Beyotime), rabbit monoclonal anti-mouse histone H3 (citrulline R17) (ab219407) (Abcam, Cambridge, UK). Finally, the distinct protein bands were detected by ECL detection reagent (Beyotime). The bands in western blot were quantified into numerical values using image J software.

### Immunofluorescent staining

PEC and neutrophils were cultured in a 48-well plate and infected with *P. multocida* for 3 h. After infection, cells were washed three times with PBS and fixed in 4% paraformaldehyde (Sango Biotech, Shanghai, China) for 20 min at room temperature (RT). After three wash steps, cells were permeabilized with 0.1% Triton X-100 in PBS for 10 min. Subsequently, cells were blocked with 5% Bovine Serum Albumin (BSA) in PBS for 30 min. Then, the cells were stained with primary antibody containing rabbit anti-mouse NLRP6 (bs-10440R) (Bioss) and mouse anti-rabbit ASC (sc-514414) (Santa Cruz, CA, USA) for 2 h at RT, rabbit monoclonal anti-mouse histone H3 (citrulline R17) (ab219407) (Abcam, Cambridge, UK). After the washing steps, cells were incubated with Goat anti-mouse IgG (H&L) Alexa fluor 488 and Goat anti-rabbit IgG (H&L) Alexa fluor 594 (Abcam, UK) for 1 h. DAPI (Beyotime Biotechnology, Shanghai, China) was added for 5 min to visualize the cell nucleus. Finally, cells were washed and maintained in antifading medium (Solarbio, Beijing, China). The cells were observed on the fluorescence microscope (Olympus, Tokyo, Japan). Image J was used to calculate the positive staining cells.

### Quantitative RT-PCR

Mice and macrophages were infected with *P. multocida* as described above. The samples (BALF and lung tissue) were collected at 3, 6, 9, and 24 h after infection and total RNA (500 ng) was extracted using the RNA Pre-Pure kit (Tiangen, Beijing, China). Then, complementary DNA was synthesized with the PrimeScript RT reagent Kit (TaKaRa, Dalian, China) according to the manufacturer’s instructions. Quantitative real-time RT PCR was carried out using SsoFast Eva Green Super-Mix (Bio-Rad, Hercules, CA, USA) and performed on a Bio-Rad CFX 96 instrument. Primers [12–16] used in this study are shown in Table 1. The reaction procedure was the following: 95 °C, 2 min; 95 °C, 5 s, 40 cycles; 60 °C, 30 s; 95 °C, 5 s; 60 °C, 5 s; 95 °C, 2 min. Three replicates were set for each gene, and 2-^ΔΔ^CT was used for relative quantitative analysis.

**Table 1:** **Primer and probe sequences for qPCR**.

Gene		5′3’sequence
***β-actin***	Forward	GTGACGTTGACATCCGTAAAGA
	Reverse	GCCGGACTCATCGTACTCC
***il-1β***	Forward	GAA ATG CCA CCT TTT GAC AGT G
	Reverse	TGG ATG CTC TCA TCA GGA CAG
***nlrp3***	Forward	ATT ACC CGC CCG AGA AAG G
	Reverse	CAT GAG TGT GGC TAG ATC CAA G
***cxcl1***	Forward	ACT GCA CCC AAA CCG AAG TC
	Reverse	TGG GGA CAC CTT TTA GCA TCT T
***cxcl2***	Forward	CCA ACC ACC AGG CTA CAG G
	Reverse	GCG TCA CAC TCA AGC TCT G
***cxcr2***	Forward	ATG CCC TCT ATT CTG CCA GAT
	Reverse	GTG CTC CGG TTG TAT AAG ATG AC

### Flow cytometric analysis

Mice were infected with *P. multocida* for 24 h as described above. After washing the BALF, the total number of cells were collected and counted. After centrifugation and washing steps, cells were blocked with 2% BSA in PBS for 30 min. First, the anti-mouse CD16/32 antibody (1:50) (BioLegend, San Diego, CA, USA) was used to block nonspecific antibody binding in 0.5% BSA buffer at 4 °C for 30 min. Then the cells were stained with PE-labeled anti-mouse F4/80 mAb (1:40) (BioLegend) and FITC-labeled anti-(Ly-6G/Ly-6C)Gr-1 mAb (1:200) (BioLegend) in 0.5% BSA buffer at 4 °C for 30 min. Afterwards, cells were washed and analyzed using flow cytometry. Novo Express was used for experimental operations and data analysis (Agilent, Beijing, China).

### Statistical analysis

Statistical analysis was performed using GraphPad Prism software v6 (San Diego, CA, USA), and the student *t*-test was used to analyze the statistical differences for comparisons between two groups. Statistical significance was determined as the *p* value, *P < 0.05, **P < 0.01, ***P < 0.001, ns = no significance.

## Results

### NLRP6 protects the lung from *P. multocida* infection

**Figure 1:**
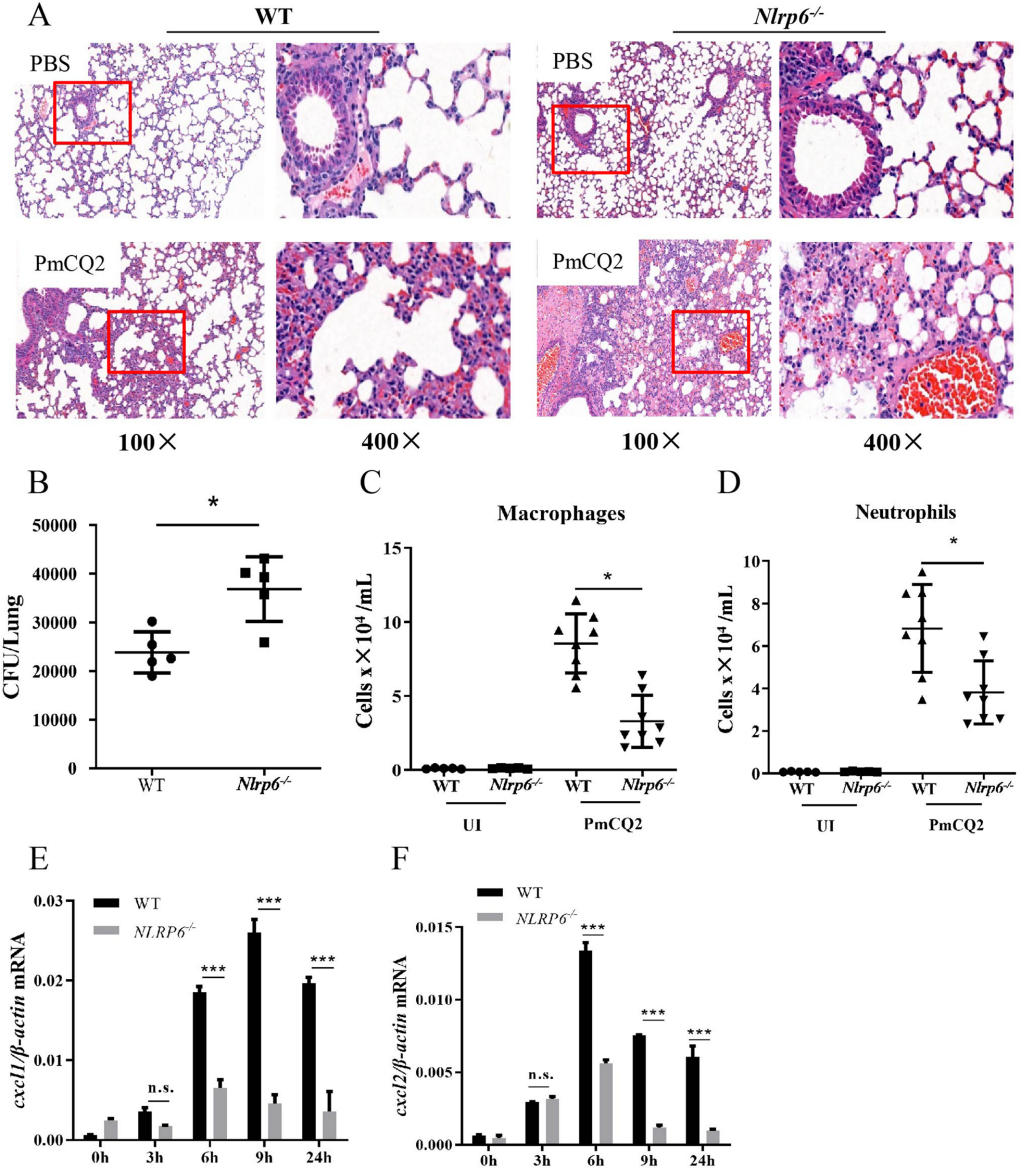
**NLRP6 protects the lung from *P. multocida* infection.** WT and *Nlrp6*^*−/−*^ mice were intranasally infected with 20 µL *P. multocida* (1000 CFU) and the same volume of sterilized PBS were used as control. Lungs were collected after 48 h infection for hematoxylin and eosin staining (original magnification × 100 and × 400). **A** Pictures are representative of three mice from each group of mice (*n* = 3). **B** The lungs were homogenized and colony counting was performed after 48 h infection (*n* = 5). Flow cytometry was used to analyze the number of neutrophils (**C**) and macrophages after 24 h infection (**D)** (*n* = 5–8). Lung homogenates were collected after 3 h, 6 h, 9 h, 24 h infection, and tissue RNA was extracted to detect mRNA expression of *cxcl1* (**E**) and *cxcl2* (**F**) mRNA (*n* = 5 in each time point). The student *t*-test was used to analyze the statistical differences for comparisons between two groups. Statistical significance was determined as *p* value, *P < 0.05, **P < 0.01, ***P < 0.001, ns: no significance.

In order to determine the role of NLRP6 in the host defense against *P. multocida* in vivo, WT and *Nlrp6*^*−/−*^ mice were infected and then lung tissues were collected for H&E staining. The results show that both lungs of *Nlrp6*^*−/−*^ and WT mice had inflammation, infected mice show a large number of inflammatory cell infiltrations and increased alveolar interstitial, indicating that *P. multocida* induced severe inflammation in the lungs (Figure 1A, red rectangle). However, *Nlrp6*^*−/−*^ mice had more bacteria in the lungs after infection compared with WT mice (Figure 1B). To determine whether bacterial clearance in the lungs was affected by the recruitment of immune cells, the number of neutrophils and macrophages was analyzed by flow cytometry after 24 h infection. The results show that the number of neutrophils and macrophages was significantly lower in the lungs of *P. multocida*-infected *Nlrp6*^*−/−*^ mice than those of WT mice (Figures 1C, D and Additional file 1). It has been reported that chemokines CXCL1 and CXCL2 play an important role in the recruitment of neutrophils and macrophages [17]. Our RT-PCR results show that *cxcl1* mRNA peaked 9 h after *P. multocida* infection, and *cxcl2* mRNA peaked 6 h after *P. multocida* infection, but the WT mice were significantly higher than those of *Nlrp6*^*−/−*^ mice (Figures 1E, F). These results indicate that NLRP6 plays a protective role in the host defense against *P. multocida*.

### NLRP6 affects the formation of neutrophil extracellular traps (NET)

**Figure 2:**
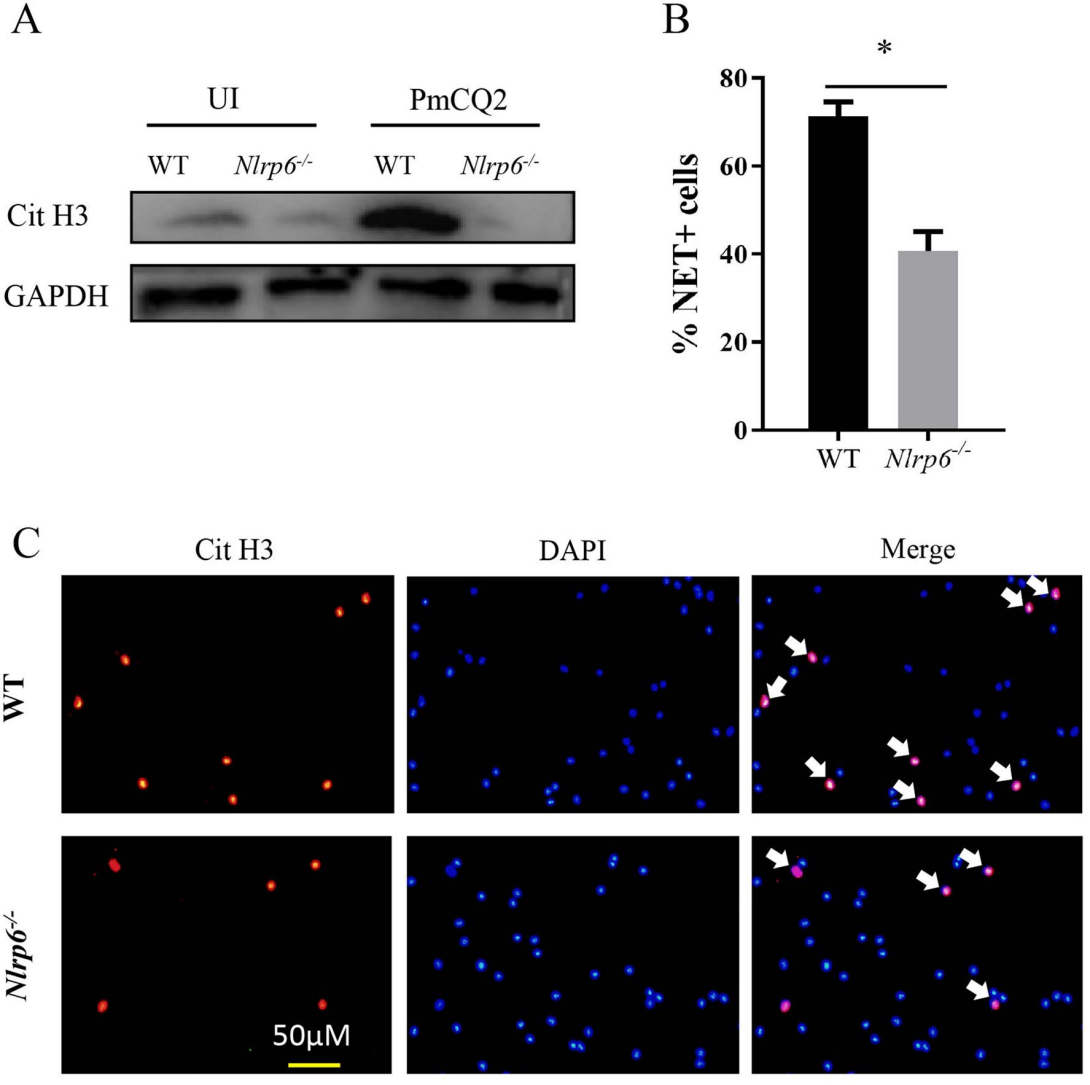
**NLRP6 affects the formation of NET.** Neutrophils from WT or *Nlrp6*^*−/−*^ mice were infected with *P. multocida* at a MOI of 1 for 3 h. The supernatants (Sup) and cell lysates (Lys) were collected after infection. Cit H3 protein expression was detected by western blot analysis and immunofluorescent staining. A representative image of Cit H3 protein expression is shown (**A**) The number of neutrophils with NET was quantified (**B**). Cit H3 protein (red) and DAPI (blue) were observed through fluorescence microscopy (**C**). The white arrow indicates the co-localization of Cit H3 and DAPI. Image J was used for quantitative analysis. The results are representative of three independent experiments. The student *t*-test was used to analyze the statistical differences for comparisons between two groups. Statistical significance was determined as *p* value, *P < 0.05, **P < 0.01, ns: no significance.

Neutrophil extracellular traps (NET) are induced by inflammatory stimuli and play a critical role in killing invading pathogens. It has been reported that modification by citrullination of histone H3 (Cit H3) is considered to be involved in the formation of NET [18]. Therefore, whether NLRP6 affects NET formation was investigated by detection of Cit H3 protein expression. The results show that the expression of Cit H3 protein was significantly decreased in *P. multocida-*infected *Nlrp6*^*−/−*^ mice (Figures 2A, B) and immunofluorescent staining results also show decreased NET formation (Figure 2C). These results suggest that NLRP6 can promote the formation of NET in *P. multocida* infection.

### NLRP6 mediates expression of inflammatory cytokines in macrophages infected with *P. multocida*

**Figure 3:**
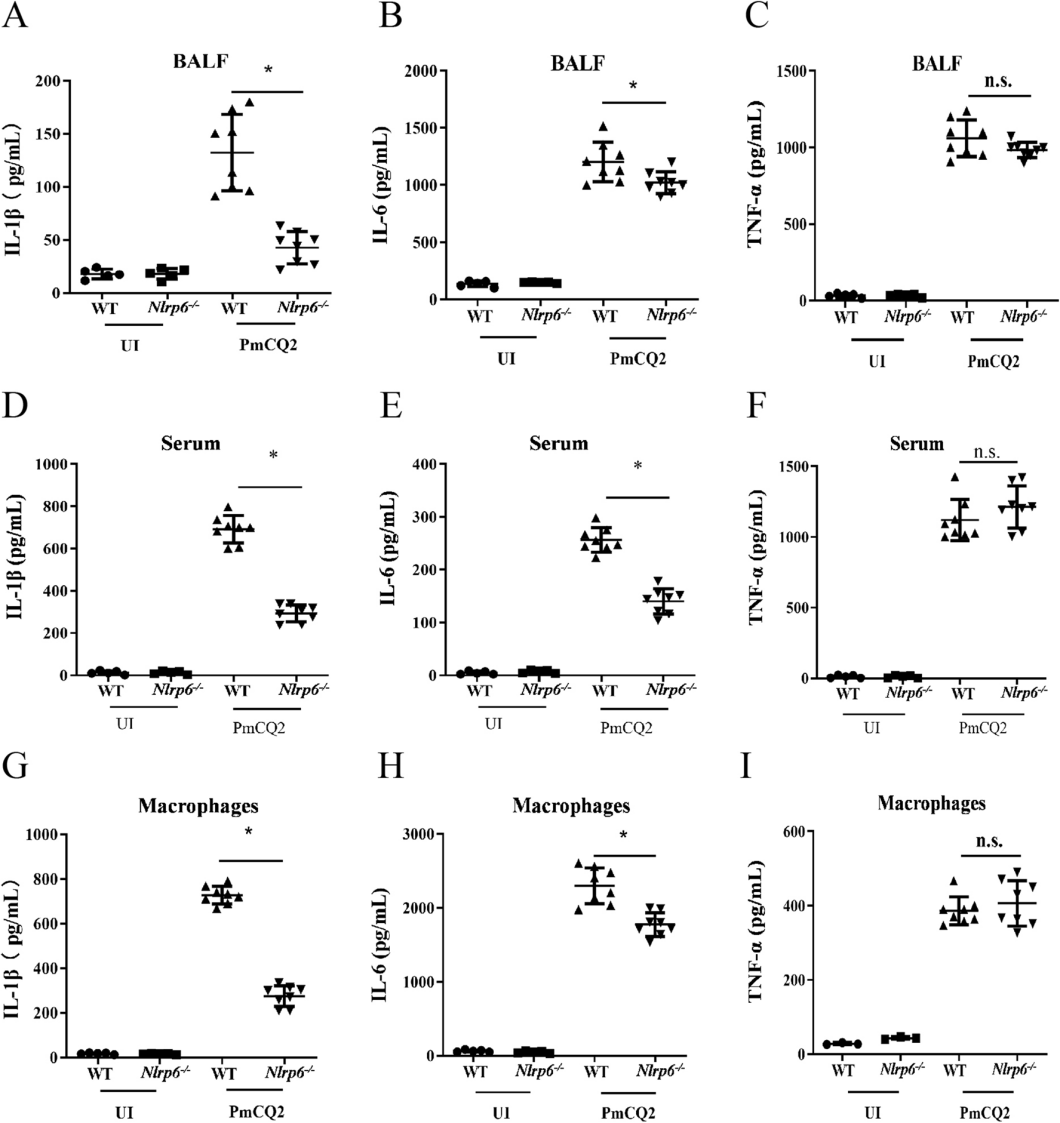
**NLRP6 mediates expression of inflammatory cytokines in macrophages infected with *P. multocida*.** WT and *Nlrp6*^*−/−*^ mice were intranasally infected with 20 µL *P. multocida* (1000 CFU) and the same volume of sterilized PBS were used as the control. Bronchoalveolar lavage fluid (BALF) and peripheral serum were collected after 48 h infection (*n* = 5–8). ELISA was used to detect the levels of IL-1β (**A**), IL-6 (**B**), TNF-α (**C**) in BALF and IL-1β (**D**), IL-6 (**E**) and TNF-α (**F**) in peripheral serum (*n* = 5–8). Macrophages from WT or *Nlrp6*^*−/−*^ mice were infected with *P. multocida* at an MOI of 1 for 9 h. Then 100 μg/mL ciprofloxacin was added for an additional 15 h incubation. After 24 h infection, the supernatants were collected, and IL-1β (**G**), IL-6 (**H**) and TNF-α (**I**) were detected by ELISA. The student’s *t*-test was used to analyze the statistical differences for comparisons between two groups. Statistical significance was determined as the *p* value, *P < 0.05, **P < 0.01, ns: no significance.

To understand the mechanism of NLRP6 resistance to *P. multocida* infection, cytokine secretion was measured in vitro and in vivo after 48 h infection. The results show that the secretion levels of IL-1β and IL-6 from the BALF and serum of *Nlrp6*^*−/−*^ mice were significantly lower than those of WT mice after infection (Figures 3A, B, C, E), while the secretion levels of TNF-α did not change (Figures 3C, F). Moreover, the expression of inflammatory cytokines was also determined in primary peritoneal macrophages of WT and *Nlrp6*^*−/−*^ mice infected with *P. multocida*. Similarly, the secretion of these cytokines in macrophages was consistent with the trend in vivo (Figures 3G, H, I). These results suggest that NLRP6 is involved in the secretion of IL-1β and IL-6.

### NLRP6 mediates caspase-1 activation and ASC oligomerization in macrophages infected with *P. multocida*

**Figure 4:**
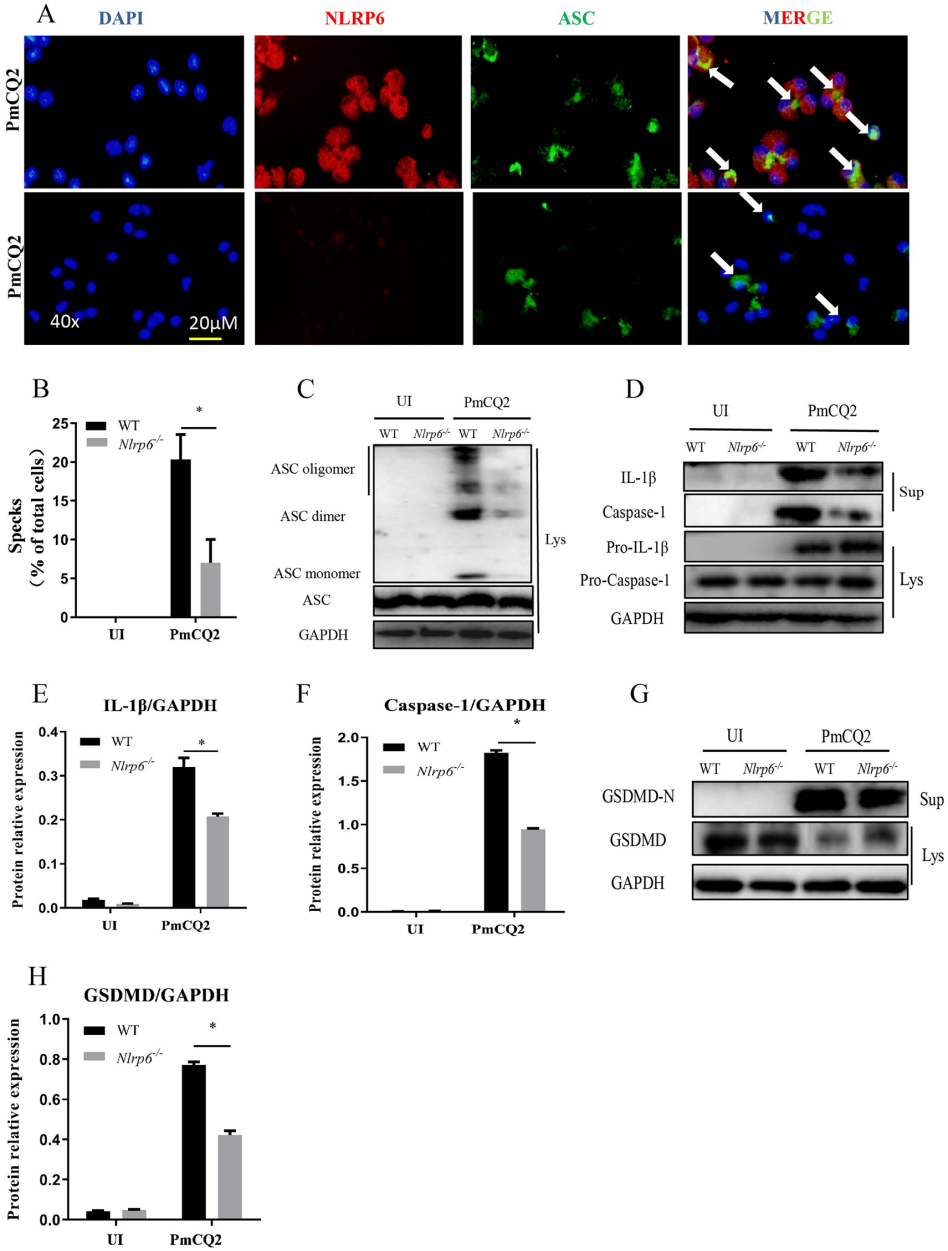
**NLRP6 mediates activation of caspase**-**1 and ASC in macrophages infected with *P. multocida*.** The peritoneal macrophages of WT mice and *Nlrp6*^*−/−*^ mice were infected with *P. multocida* (1.3 × 10^5^ CFU) for 3 h. NLRP6 protein (red) and ASC specks (green) were observed through fluorescence microscopy (**A**). The white arrow indicates the co-localization of NLRP6 and ASC. The number of ASC specks in *P. multocida*-infected WT and *Nlrp6*^*−/−*^ mice macrophages was quantified (**B**). Macrophages from WT or *Nlrp6*^*−/−*^ mice were infected with *P. multocida* at an MOI of 1 for 9 h. The supernatants (Sup) and cell lysates (Lys) of WT and *Nlrp6*^*−/−*^ mice peritoneal macrophages infected with *P. multocida* were collected after 24 h infection. Western blot analysis was used to detect the ASC oligomerization (**C**) and secretion of IL-1β (p17: subunit; p31: precursor), caspase-1 (p20: subunit; p45: precursor) (**D**) as well as GSDMD (**G**). Image J was used to quantify the ratio of IL-1β (**E**), caspase-1 (**F**), and GSDMD-N (**H**) to GAPDH. The results are representative of three independent experiments. Student’s *t*-test was used to analyze the statistical differences for comparisons between two groups. Statistical significance was determined as *p* value, *P < 0.05, **P < 0.01, ns: no significance.

To further assess the mechanism of *P. multocida*-induced NLRP6 activation, we investigated the activation of caspase-1, formation of ASC speck and ASC oligomerization in macrophages. The results of immunofluorescent staining show that ASC speck (Figure 4A, white arrows) was induced by *P. multocida* and co-localized with NLRP6 protein but NLRP6 knock out significantly decreased ASC speck in macrophages (Figures 4A, B). Furthermore, ASC oligomerization was also induced by *P. multocida* in WT macrophages, but not in *Nlrp6*^*−/−*^ macrophages (Figure 4C). These results suggest that NLRP6 inflammasome formation and activation are associated with the recruitment of ASC to NLRP6. It has been reported that the adaptor ASC can regulate caspase-1 activation and promote the secretion of pro-inflammatory cytokines IL-1β [19]. Our results show that *P. multocida* induced the secretion of IL-1β and caspase-1 while their secretion was significantly reduced in *Nlrp6*^*−/−*^ macrophages, but the expression of precursors pro-IL-1β and pro-caspase-1 was not affected (Figures 4D, E, F), indicating that *P. multocida*-induced caspase-1 activation requires the formation of NLRP6 inflammasome.

In addition to inducing inflammatory cytokine expression, microbial infection also induces pyroptosis. GSDMD is an important factor to form pores on the membrane and induce pyroptosis. Anand et al. showed that NLRP6 can affect the expression of GSDMD under the stimulation of *streptococcus pneumoniae* [20]*.* Therefore, to examine whether NLRP6 involved in *P. multocida*-induced pyroptosis, GSDMD maturation was investigated. The results show that *Nlrp6*^*−/−*^ mouse macrophages secreted less GSDMD than WT macrophages during infection (Figures 4G, H), which demonstrates that NLRP6 is involved in the modulation of pyroptosis.

### NLRP6 is involved in the expression of NLRP3 in *P. multocida*-infected macrophages

**Figure 5:**
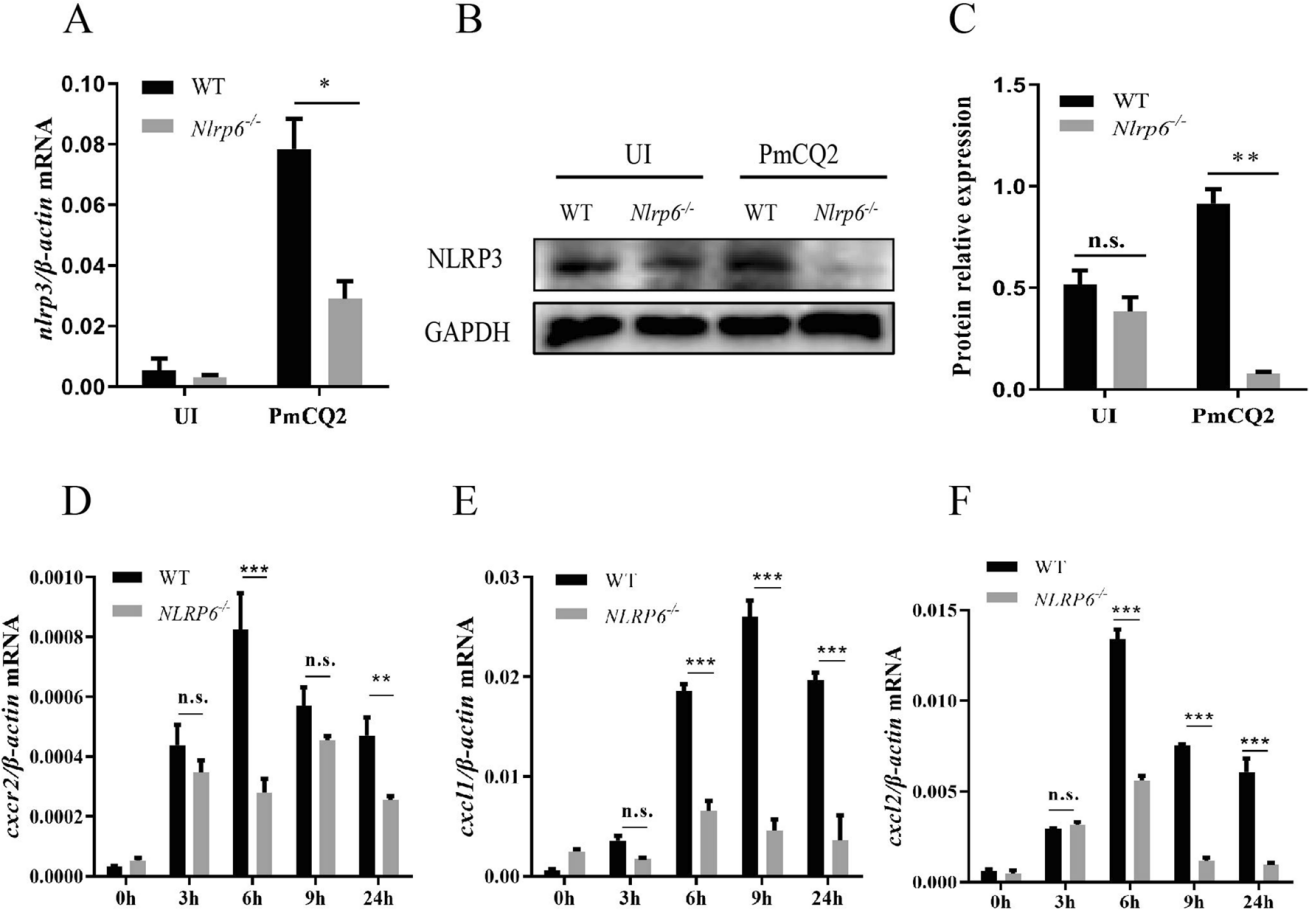
**NLRP6 is involved in the expression of NLRP3 in *P. multocida* infected macrophages.** Macrophages were infected with the *P. multocida* at a MOI of 1 for 9 h. Then 100 μg/mL ciprofloxacin was added for an additional 15 h incubation. After 3, 6, 9 and 24 h incubation, cell lysates were collected for RT-PCR and western blot assays. *nlrp3* mRNA expression was determined by RT-PCR (**A**). NLRP3 protein expression was detected by western blot (**B**) and the ratio of NLRP3 level against GAPDH level was quantified (**C**). *P. multocida*-infection after 3, 6, 9 and 24 h detected relative mRNA expression of *cxcr2* (**D**), *cxcl1* (**E**), and *cxcl2* (**F**) were also determined using RT-PCR. The results are representative of three independent experiments. Student *t*-test was used to analyze the statistical differences for comparisons between two groups. The statistical significance was determined as the *p* value, *P < 0.05, **P < 0.01, ***P < 0.001, ns: no significance.

According to the study by Anand et al., the lack of NLRP6 up-regulates the expression of NLRP3 in liver tissues [20]. Our previous study showed that *P. multocida* also activates NLRP3 and leads to IL-1β secretion [21]. Therefore, NLRP3 expression was detected to investigate whether NLRP6 affects *P. multocida*-induced NLRP3 activation in macrophages. The results show that the expression level of both NLRP3 protein and *nlrp3* mRNA was significantly downregulated in *P. multocida*-infected *Nlrp6*^*−/−*^ macrophages (Figures 5A, B, C). Similarly, mRNA expression of chemokines *cxcl1*, *cxcl2* and CXC chemokine receptor 2 (*cxcr2*) which drives the activation of NLRP3 inflammasomes [22], was also significantly decreased in *P. multocida*-infected *Nlrp6*^*−/−*^ macrophages (Figures 5D, E, F) at 3, 6, 9, and 24 h post-infection. These results suggest the possible modulatory role of NLRP6 in the formation of NLRP3 inflammasome by inducing the expression of chemokines.

## Discussion

*P. multocida* is a highly virulent pathogen causing serious respiratory infection in different animals including cattle, pigs and chickens. So far, the treatment of *P. multocida* mainly relies on antibiotics but the use of antibiotics contributes to the development of resistance [23]. Therefore, new therapies are needed to treat *P. multocida* infection. Understanding the host innate immune response mechanism against *P. multocida* will promote the manipulation of host signaling events for the design of potential therapeutic interventions. Inflammasomes are a part of the innate immunity system and play an important role in the host defense against microbial infection. NLRP6 inflammasome was recently identified and shown a protective role in the intestine where it is highly expressed [24]. Interestingly, NLRP6 is also highly expressed in the respiratory tract but it plays a negative role in the lungs against gram-positive bacterial infection [8, 20, 25]. Despite the multiple roles of NLRP6 in the host, its role in gram-negative bacterial pulmonary infection is rarely studied. Therefore, we investigated the role of NLRP6 in the lungs and NLRP6 inflammasome activation in response to *P. multocida*.

Due to the high expression of NLRP6 in intestinal epithelial cells, initial studies have focused on its role in intestinal disease. For example, NLRP6 has been shown to regulate intestinal antiviral innate immunity [26]. Like the detection of NLRP6 expression in immune cells including neutrophils, macrophages and dendritic cells, the role of NLRP6 in lung inflammation has also been studied extensively. NLRP6 knockout mice were found to have less bacterial burden in the lungs or BALF and show less severe lung injury in response to different bacterial infection, such as *Staphylococcus aureus*, *Listeria monocytogenes*, *Salmonella typhimurium* and *Streptococcus pneumoniae* [8, 20, 27]*.* Furthermore, the negative role of NLRP6 in *Staphylococcus aureus* and *Streptococcus pneumoniae* infection was found to be regulated by neutrophil recruitment as a higher number of neutrophils was detected in bacterial infected-*Nlrp6*^*−/−*^ mice [8, 25]. However, NLRP6 showed a critical role in response to *Klebsiella pneumoniae* infection as NLRP6 knock out significantly reduced the recruitment of neutrophils and the formation of NET [28]. Our data show that *Nlrp6*^*−/−*^ mice have severe inflammation in the lungs and a higher bacterial burden in BALF as well as a lower number of macrophages and neutrophils. Furthermore, NLRP6 negatively modulated the formation of NET. These results indicate the positive role of NLRP6 in the lungs against *P. multocida*, which is consistent with the findings that NLRP6 is a positive regulator in *Klebsiella pneumoniae-*induced pneumonia [28]. In addition, our study shows that NLRP6 mediates the expression of chemokines CXCL1 and CXCL2. It has been reported that these chemokines are involved in neutrophil recruitment [29]. Therefore, the protective role of NLRP6 is likely exerted via chemokine expression, but the mechanism of chemokine-mediated NLRP6 inflammation needs to be further studied. However, NLRP6 activation induced by *Staphylococcus aureus*, *Listeria monocytogenes*, *Salmonella typhimurium* and *Streptococcus pneumoniae* results in excessive inflammation, likely indicating that NLRP6 plays a critical role in amplifying inflammatory response during these microbial infections.

Similarity with NLRP3 inflammasome activation, NLRP6 inflammasome activation results in IL-1β secretion. Hara et al. found that NLRP6 is involved in the secretion of IL-1β, IL-18, and IL-6 in bone marrow derived macrophages (BMDM) infected with *Listeria monocytogenes* and *Staphylococcus aureus* [30]. Other previous studies also showed attenuated secretion of IL-1β and IL-6 in *Nlrp6*^*−/−*^ mouse macrophages infected with S*treptococcus pneumoniae* [25, 31]. Similarly, our present study shows that NLRP6 modulates IL-1β and IL-6 secretion both in vitro and in vivo in response to *P. multocida* infection. Despite the reduced IL-1β secretion in *Nlrp6*^*−/−*^ mice or cells, Anand et al. found that NLRP6 knockout increases another inflammatory cytokine IL-6 secretion in response to *Listeria monocytogenes* infection [20, 32]. While NLRP6 knockout attenuates IL-6 secretion during *Streptococcus pneumoniae* and *P. multocida*, it is likely that NLRP6-mediated IL-1β and IL-6 secretion may exhibit different signaling pathways in response to different pathogen infections, and the exact mechanism of NLRP6-mediated IL-6 secretion needs to be further explored. In addition, Chen et al. found that NLRP6 also mediated gene expression of TNF-α in dextran sulfate sodium (DSS)-induced colitis and tumorigenesis [33]. However, our study shows that TNF-α secretion is not altered in *Nlrp6*^*−/−*^ macrophages, indicating that PmCQ2-induced TNF-α might not be involved in the development of tumorigenesis as this cytokine is tumor promoting.

Inflammasome regulates the activation of caspase-1, which process pro-IL‐1β into active maturation of IL-1β and also induces the activation of GSDMD [19]. During this process, the connector molecule ASC plays an important role in caspase-1 and caspase-11 recruitment [34]. Our study suggests that the NLRP6 inflammasome activation induced by *P. multocida* promotes caspase-1 and GSDMD activation. Furthermore, *P. multocida* induces the formation of ASC specks and ASC-NLRP6 co-localization in *P. multocida-*infected macrophages, which is similar to the observation that NLRP6 co-localizes with ASC and caspase-1 to form NLRP6 inflammasome resulting in cytokine secretion in intestinal epithelial cells [35].

Our previous study showed that *P. multocida* induces NLRP3 inflammasome activation [21]. Interestingly, NLRP6 knockout abrogates NLRP3 protein expression in *P. multocida*-infected macrophages, suggesting that a synergy effect of NLRP3 and NLRP6 in the host. In contrast, Li et al. reported that the expression of NLRP3 in the liver of *Nlrp6*^*−/−*^ mice is significantly higher than that of WT mice under liver injury conditions while Cai et al. found that NLRP6 does not affect NLRP3 expression in response to *Klebsiella pneumoniae* infection [28, 36]. Furthermore, recombinant CXCL1 can rescue host survival, bacterial clearance, neutrophil influx in *Klebsiella pneumoniae*-infected *Nlrp6*^*−/−*^ mice, indicating the important role of CXCL1 in the host. In this study, NLRP6 knockout resulted in decreased secretion of IL-1β and IL-6, the expression of CXCL1 and CXCL2 increased and then decreased with time, and there was a decreased recruitment of macrophages and neutrophils during *P. multocida* infection. Therefore, we reasonably speculate that NLRP6 modulates the expression of CXCL1 and CXCL2, thereby regulating the expression of NLRP3 and the influx of immune cells, but the specific situation needs to be verified.

In summary, *P. multocida* activated NLRP6 inflammasome with the formation of ASC specks, which mediated caspase-1 activation and ASC oligomerization, resulting in IL-1β secretion. Furthermore, NLRP6 regulated activation of NLRP3 inflammasome may be via chemokines CXCL1, CXCL2, and CXCR2. Finally, NLRP6 shows a protective role in the host defense against *P. multocida* infection. Our study provides new insights into NLRP6 inflammasome-mediated inflammation in the host defense against *P. multocida* infection.

## Supplementary Information

**Additional file 1: NLRP6 is involved in the recruitment of macrophages and neutrophils in pulmonary infection with *P. multocida*. **WT and *Nlrp6*-/- mice were intranasally infected with 20 µL *P. multocida *(1000 CFU) and the same volume of sterilized PBS were used as control. Flow cytometry was used to analyze the recruitment of neutrophils and macrophages after 24 h infection. (A, B, E, F). Aseptic PBS infection group (control group). (C, D, G, H). *P. multocida *infection group (infection group). The results are representative of three independent experiments and the trend was consistent each time.
