# Supplementary figures and images for: The important role of NLRP6 inflammasome in Pasteurella multocida infection
Source: Vet Res. 2022 Oct 12;53:81. doi: 10.1186/s13567-022-01095-0 (PMC9558406; doi:10.1186/s13567-022-01095-0)

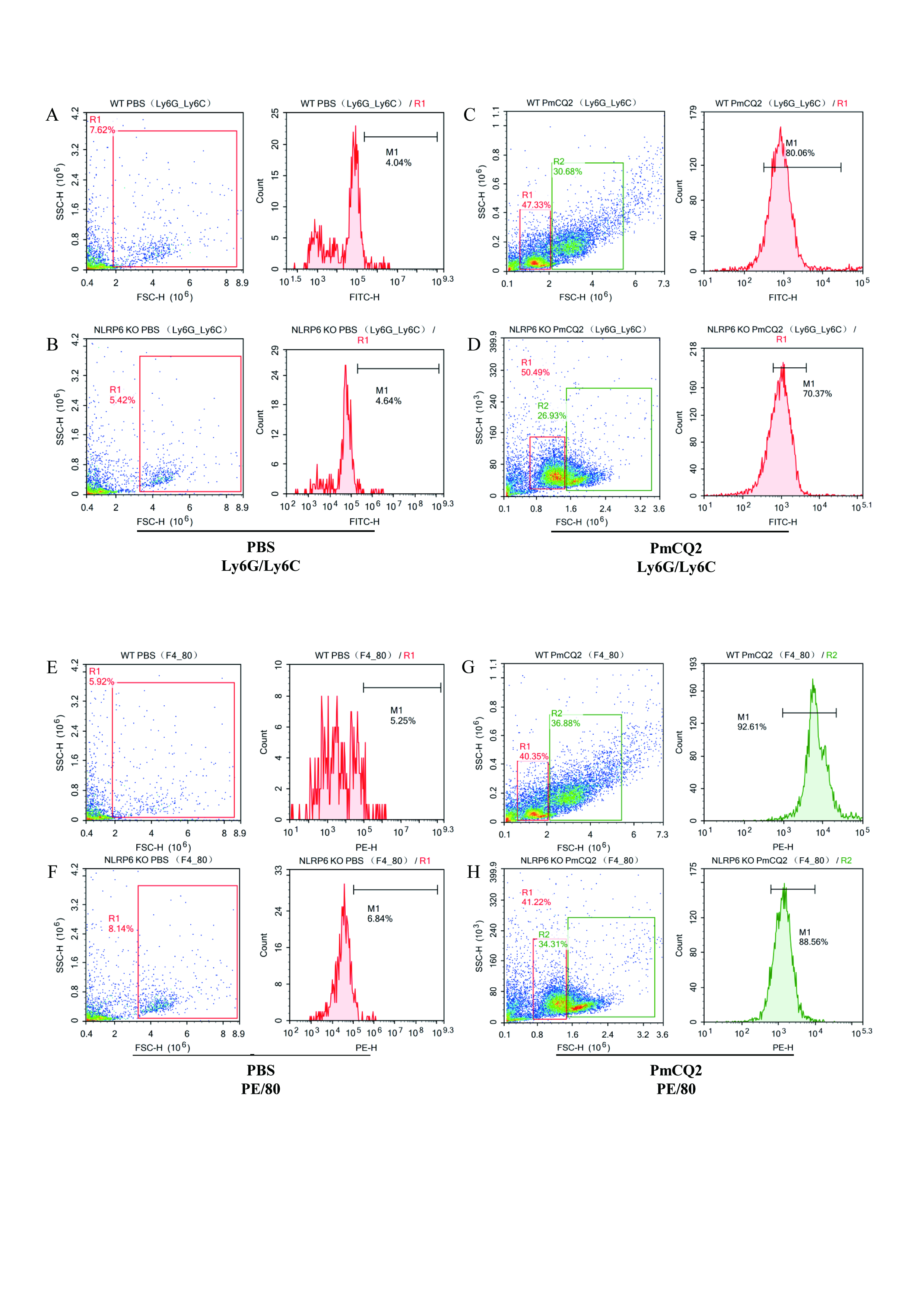

Supplement: Supplementary file 1 — Additional file 1: NLRP6 is involved in the recruitment of macrophages and neutrophils in pulmonary infection with P. multocida. WT and Nlrp6-/- mice were intranasally infected with 20 µL P. multocida (1000 CFU) and the same volume of sterilized PBS were used as control. Flow cytometry was used to analyze the recruitment of neutrophils and macrophages after 24 h infection. (A, B, E, F). Aseptic PBS infection group (control group). (C, D, G, H). P. multocida infection group (infection group). The results are representative of three independent experiments and the trend was consistent each time. [file 13567_2022_1095_MOESM1_ESM.tif]
